# Computational Study of the Electron Spectra of Vapor-Phase Indole and Four Azaindoles

**DOI:** 10.3390/molecules26071947

**Published:** 2021-03-30

**Authors:** Delano P. Chong

**Affiliations:** Department of Chemistry, University of British Columbia, 2016 Main Mall, Vancouver, BC V6T 1Z1, Canada; chong@chem.ubc.ca

**Keywords:** excitation spectra, photoelectron spectra, core electron binding energies, X-ray emission spectra, indole and azaindoles

## Abstract

After geometry optimization, the electron spectra of indole and four azaindoles are calculated by density functional theory. Available experimental photoemission and excitation data for indole and 7-azaindole are used to compare with the theoretical values. The results for the other azaindoles are presented as predictions to help the interpretation of experimental spectra when they become available.

## 1. Introduction

Indole is a bicyclic aromatic compound consisting of a six-membered benzene ring fused to a five-membered pyrrole ring. Azaindoles are analogous molecules in which the benzene ring is replaced by a pyridine ring. Indole and azaindoles are involved in many biochemical reactions, as evidenced by papers, reviews [1,2,3,4], and books [5,6], in addition to popular internet sources, such as Wikipedia and Encyclopedia Britanica. While indole and 7-azaindole have been studied by several workers [7,8,9,10,11,12,13,14,15,16,17,18,19,20,21,22,23,24,25,26,27,28,29,30,31,32,33,34], the other azaindoles have received much less attention. The vibrations of indole were investigated by Collier [9], and by Walden and Wheeler [10]. The experimental rotational constants reported by Suenam et al. [11], Caminati and Bernardo [12], Gruet et al. [13], Nesvadba et al. [14], and by Vavra et al. [15] are more useful for the present study in guiding our choice of method of geometry optimization. The published data of UV absorption spectra [16,17,18,19,20,21,22,23,24,25,26,27,28,29,30,31,32,33,34] often covered different excited states. Serrano-Andres and Roos [22] used results from multi-configuration second-order perturbation theory for complete active space (CASPT2) to compare with the various observations. Similarly, Serrano-Andres and coworkers [33] used CASPT2 to study the excitations of 7-azaindole. We shall bring the comparison more up to date. Finally, the more recent experimental photoelectron spectra of indole [35] will be used to confirm the theoretical methods based on density functional theory (DFT) we have developed and tested.

## 2. Methods

Geometry optimization of indole and the four azaindoles are performed using the Gaussian package [36]. The results are summarized in Table 1. The optimized Cartesian coordinates of the five molecules can be found in the Supplementary Materials.

In our earlier studies, we used molecular geometry determined experimentally when available; otherwise, the Hartree–Fock method was often used for optimization; and MP2 and CCSD were also used occasionally. Typical dependence of calculated core-electron binding energies (CEBEs) on molecular geometry is shown in Appendix A. The molecule formaldehyde is rather rigid, whereas hydrogen peroxide is quite flexible. The results presented in Table A1 and Table A2 indicate that the dependence of CEBEs on geometry is quite small in both cases. The results of geometry optimization by B3LYP/6-31G(d) agree with the experimental rotational constants of indole and 7-azaindole so well that we decided to use B3LYP/6-31G(d) for the other three azaindoles as well.

The methods we use for computation of electron spectra have been presented several times in previous studies. After geometry optimization, we use the Amsterdam Density Functional (ADF) package [37] to calculate the various electron spectra. For vertical ionization energies (VIEs) of valence electrons, we used Method (a) = ΔPBE0(SAOP)/et-pVQZ [38], which means the energy difference calculated with the parameter-free Perdew–Burke–Ernzerhof exchange-correlation functional using the electron density obtained with the exchange-correlation potential (V_xc_) known as statistical averaging of orbital potentials (SAOP). The efficient even-tempered basis set of polarized valence quadruple-zeta (et-pVQZ) Slater-type orbitals [39] is available in the ADF package. This method has been used in many molecules [40,41,42,43,44,45,46,47,48,49,50]. The results are summarized in Table 2.

The AADs are somewhat arbitrary because they depend on the number of VIEs included. Moreover, some earlier experimental VIEs may be in error by as much as 0.1 eV because of calibration and/or overlapping of bands. In any case, the AADs are less than 0.2 eV for many molecules. Alternative methods giving reliable VIEs are symmetry-adapted cluster configuration interaction (SAC-CI) of Nakatsuji [51] and renormalized partial third-order (P3+) method of Ortiz [52]. Both methods are available in recent versions of the Gaussian package. However, SAC-CI is computationally demanding and does not seem to be preferred by photoelectron spectroscopists. On the other hand, P3+ is limited to outer-valence electrons only, whereas our Method (a) above can handle inner-valence electrons with little or no difficulties.

For reliable prediction of CEBEs, there are several points to consider: (1) the electrons of the core-hole cation are attracted by a shielded nucleus very different from that of the neutral parent, so that the basis set must be flexible enough to account for the difference. Usual basis sets of Gaussian-type orbitals (GTOs) contain a single contraction for the 1s orbital and is not flexible. In our earlier study using contracted GTOs [53], we used exponent scaling factors to solve the problem, in addition to testing the use of correlation-consistent core-valence basis sets. More recently, Bellafont et al. [54,55,56,57,58] used augmented Partridge basis sets of uncontracted GTOs to avoid the difficulty. On the other hand, the problem does not exist when we use Slater-type orbitals (STOs) in the ADF program. More often than not, we use the efficient et-pVQZ basis set [39], which contains double-zeta core basis functions in addition to effectively polarized quadruple-zeta basis functions for valence electrons.

(2) For the prediction of CEBEs, relativistic effects influence the accuracy of the calculated nonrelativistic results. In 1995 [59], we decided to use the formula (1):

I_rel_ = I_nr_ + C_rel_, with C_rel_ = K I_nr_^N^ (1)

to estimate the small relativistic correction to the calculated nonrelativistic CEBEs of C to F. The parameters K and N were obtained by fitting the difference between Pekeris’ accurate relativistic and nonrelativistic ionization energies of two-electron ions [60]. For both C_rel_ and I_nr_ in electron volts, K = 2.198 × 10^−7^ and N = 2.178. In 2005, Maruani et al. [61] reported results of Dirac-Fock correction to the ionization energies of atoms Li to Xe. The allometric fit for the Be to Ne series gave K = 6.55 × 10^−7^ and N = 2.0569. More recently, Bellafont et al. [54,55,56,57,58] also examined relativistic effects by the Dirac–Fock method on B to F atoms, with very different results. Table 3 summarizes all these efforts.

(3) Since the method we developed is based on DFT, there is the question of choice of functional. For CEBEs of B to F [53], we use the formula:

Method (b) = ΔPW86-PW91/et-pVQZ + C_rel_, (2)

which means that PW86 and PW91 are used for exchange and correlation functionals, respectively. Table 4 compares our results for carbon-containing molecules with those in the resent paper from Illas’ laboratory [17] using the TPSS functional.

Experimental measurements of CEBEs by X-ray photoelectron spectroscopy require calibration and reported values may be off by as much as 0.1 eV. As a rule, synchrotron measurements tend to be more reliable. In any case, our AAD of 0.14 eV is remarkably small. The relativistic correction for carbon 1s ionization is 0.05 eV for our method and 0.13 eV for the method using TPSS. Whether or not we add 0.08 eV to our results, our correlation corrected results for carbon-containing molecules are definitely superior to those using the TPSS functional. Table 5 compares the CEBEs for N, O, and F, calculated by our Method (b) with the results obtained by Bellafont et al. using TPSS [56].

Two other approaches tested by Illas and coworkers do not fare any better: The popular functional B3LYP performs fairly well for CEBEs of N1s but much less well for O1s [55]. Low-order GW approximations did much worse [62].

For excitation of valence electrons, we use Method (c) = time-dependent density functional theory (TDDFT) with: V_xc_ = SAOP.

Basically, TD-DFT in the ADF package is a CI calculation with singly excited configuration using DFT ground-state molecular orbitals. For non-relativistic closed shell molecules, spin and symmetry are conserved. In other words, excited singlets and triplets are computed separately. Excitations to triplet states are omitted when the keyword *allowed* is included in the input. For highly excited states (approaching Rydberg excitations, for example), the basis set et-pVQZ can be augmented by diffuse functions specially designed for excitation studies [63]. Such an augmented set (called aug-et-pVQZ) was tested on the first 15 excited states of ten closed shell molecules, and employed in the present study. The use of TDDFT for visible/UV excitations is less well validated, partly because there are fewer experimental data available for comparison. More often than not, the observed absorption bands are the result of convolution of several close-by excitations. Some comparisons between TDDFT results and experiment have been reported [63,64].

Two minor extensions are introduced in this study. Firstly, in the early days of X-ray photoelectron spectroscopy (XPS), Gelius [65,66] estimated relative cross-sections with a simple model. Minor refinements were made by Nefedov et al. [67]. The model worked remarkably well in connection with the semiempirical HAM/3 molecular orbital method [68]. The results of using such a model HAM/3 method for indole are included in Table 9, to be compared with experimental XPS of the valence electrons when available.

The second extension is called shifted meta-Koopmans’ theorem, to be used in CEBE calculations. For valence electron ionization of organic and other small molecules, meta-Koopmans’ theorem (mKT) means using the negative of the orbital energy from V_xc_ = SAOP calculation to approximate the ionization energy [69]. However, mKT does not provide reliable CEBEs, although it gives quite reasonable relative CEBEs. When there are many carbon atoms, for example, in a molecule, the core-hole may be difficult to localize. In such cases, we can obtain good estimates of the CEBEs by the method of shifted mKT. The shift needed may be obtained in various ways by comparing the mKT value with that from method (b) outlined above. In this work, we select the simplest choice for the lowest CEBE for the element of interest (carbon, for example). In the present study, the shift for indole is found to be 16.67 eV. Alternative choices for the shift are the highest CEBE (for carbon for example), the average of the lowest and highest CEBEs, or the average of all the available CEBEs.

Since the non-resonant Kα X-ray emission spectra (**XES**) are so simple to compute [70], we calculate the predicted XES for the nitrogen cores of indole and the four azaindoles. As there are so many carbon atoms in indole and azaindoles, the X-ray emission spectra are expected to be hopeless to untangle and are therefore not calculated.

## 3. Results and Discussion

The results of our calculations can of course be directly compared with experimental measurements of electron spectra. Correlation of CEBEs with Hammett substitution constants [71] has been demonstrated, but is limited to aromatic substitution reactions. In addition, Thomas et al. [72], suggested that CEBEs are related to chemical properties such as electronegativity, acidity, basicity, proton affinities, reactivity, and regioselectivity of reactions. For example, Saethre et al. [73] found statistical correlation between CEBEs and activation energies and regioselectivity for the Markovnikov addition of the elctrophiles HX (X = F, Cl, Br, and I) to the alkenes ethene, propene and 2-methylpropene. However, comparison with general experimental reactivity is much more difficult. In general, reactivity is affected by a combination of several physical properties of the reactants. For example, it was suggested that the biological activity of non-steroidal anti-inflammatory drugs (NSAIDs) depends on a synergistic collective action of several factors, including the ionization energies of molecular orbital localized mainly on the group responsible for bridging to the receptor [30].

The UV absorption of indole and 7-azaindole are compared in Table 6 and Table 7.

**Figure 1:**
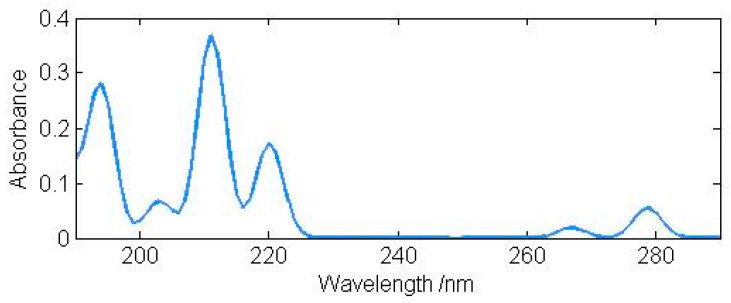
Calculated UV absorption spectrum of indole (g).

The result for indole is displayed in Figure 1. Our DFT results for the four lowest excited singlet states agree with the previous CASPT2 results reasonably well. For the higher singlet states, our DFT method appears to be more reliable. The same method and basis set are then used to predict the excited states of 4-azaindole, 5-azaindole, and 6-azaindole. Hopefully, the results, summarized in Table 8, will be useful to experimental chemists. For indole and the four azaindoles, π-type excitations have low intensities.

The results for the photoelectron spectrum of indole(g) are summarized in Table 9. Plekan et al. [35] used the method of renormalized partial third order method (P3+) for the ionization energies of outer valence electrons and ΔB3LYP for core electrons. Besides the inclusion of inner-valence electrons, our DFT results appear to be more reliable (except for the lowest VIE) than the P3+ method, especially for core electrons.

The same procedures are applied to the azaindoles and the results are shown in Table 10. The iterations for four valence cations fail to converge. For the purpose of computing X-ray emission spectra, the ionization energies of those four non-convergent cases are estimated.

Finally, the Kα X-ray emission spectra for decay of N1s core holes are predicted for indole and the four azaindoles. The results summarized in Table 11; Table 12 indicates that ΔE values are fairly similar with many transitions between 374 and 398 eV. However, the data of f-values (even though approximate) help to make the spectra different from one another. The f-values have been calculated with Kohn–Sham orbitals from V_xc_ = SAOP calculations. In atomic units, the energy difference ΔE enters the formula for f-value

f = (2/3) (ΔE)∣μ∣ ^2^ (3)

where μ is the transition dipole moment. The error in (ΔE) amounts to about 5% (20 eV in 400 eV) for nitrogen. Therefore, the *f*-values listed are hardly affected except for the most intense transitions, which have been highlighted with boldface type in Table 11 and Table 12.

## 4. Summary

In this work, we have computed the various electron spectra of gas-phase indole and four azaindoles. The spectra include UV absorption, valence ionization, core ionization, and X-ray emission. The available experimental data on indole and 7-azaindole allow the comparison of theory with experiment and support the reliability of the predicted spectra. Experimentalists are therefore encouraged to measure the unknown spectra.

## Figures and Tables

**Table 1:** Experimental (microwave) and theoretical (present work) rotational constants (in MHz) of indole and azaindoles.

Year	Indole	A	B	C	AAD ^a^	Dipole (ADF) ^b^, D	α ^b^	Δα ^b^
1988	Suenram et al. [11]	3877.84	1636.05	1150.90				
1990	Caminati and Bernardo [12]	3877.83	1636.05	1150.90		2.09 ± 0.13		
2015	Gruet et al. [13]	3877.84	1636.05	1150.90				
2017	Nesvadba et al. [14]	3877.84	1636.05	1150.90				
2019	Vavra et al. [15]	3877.84	1636.05	1150.90	(0)			
2020	B3LYP/6-31G(d)	3878.25	1631,22	1148.25	2.63	2.1731 (2.1798)	103.35	118.93
2020	B3LYP/6-311+G(2d,p)	3901.39	1640.17	1154.72	10.50	2.1360		
2020	B3LYP/cc-pVTZ	3908.14	1642.93	1156.68	14.32	2.1732		
2020	B3LYP/cc-pVQZ	3909.46	1643.62	1157.14	15.14	2.1399		
**7-Azaindole**								
1990	Caminati and Bernardo [12]	3928.93	1702.63	1188.13	(0)	1.68 ± 0.07		
2020	B3LYP/6-31G(d)	3929.63	1697.39	1185.37	2.90	1.6343 (1.7166)	97.99	147.73
2020	B3LYP/6-311+G(2d,p)	3957.32	1705.05	1191.16	11.28	1.6435		
2020	B3LYP/cc-pVTZ	3963.53	1708.56	1193.91	15.44	1.6198		
2020	B3LYP/cc-pVQZ	3966.63	1708.97	1194.38	16.76	1.6293		
**4-Azaindole**								
2020	B3LYP/6-31G(d)	3914.94	1692.94	1181.87		4.0322 (4.1588)	98.13	127.01
**5-Azaindole**								
2020	B3LYP/6-31G(d)	4014.22	1638.31	1163.47		4.4053 (4.5675)	97.05	90.37
**6-Azaindole**								
2020	B3LYP/6-31G(d)	4012.95	1639.08	1163.75		3.7656 (3.9394)	97.09	122.91

^a^ Average absolute deviation. ^b^ From SAOP/aug-et-pVQZ.

**Table 2:** Vertical ionization energies (in eV) of valence electrons calculated ΔPBE0 (SAOP). AAD = Average absolute deviation from experiment.

Ref.	Year	Molecules	Number of VIEs	AAD, eV
38	2009	31 molecules	128	0.26
40	2010	*S*-triazine	10	0.20
41	2010	naphthalene	19	0.15
		azulene	9	0.13
42	2010	1,4-benzoquinone	16	0.19
43	2011	formamide	9	0.11
44	2011	cyclopentadiene	11	0.26
		pyrrole	10	0.19
		furan	12	0.23
		thiophene	11	0.27
45	2012	2,1,3-benzothiadiazole	10	0.14
		1,3,2,4-benzodithiadiazine	10	0.09
		1,3,5,2,4-benzodithiadiazepine	11	0.15
46	2013	5-methyltetrazole	9	0.22
47	2013	uric acid	5	0.14
48	2017	acetamide	9	0.17
		*N*-methylformamide	8	0.16
49	2019	dimethylnitrosamine	5	0.13
		*N*-nitrosopyrrollidine	6	0.16
		1-nitrosoaziridine	9	0.24
50	2019	Pyridine	12	0.15
		1,2-diazine	13	0.28
		1,3-diazine	12	0.25
		1,4-diazine	12	0.25

**Table 3:** Relativistic correction of core electron ionization energies (in eV).

Case	Ref	Effects Included				B	C	N	O	F
		Relativistic	Correlation	Molecular						
Two Electron Ions	60	yes	yes	no	I_rel_	259.375	392.093	552.072	739.335	953.911
					I_nr_	259.338	391996	551.865	738.944	953.234
					I_rel_–I_nr_	0.037	0.097	0.207	0.391	0.677
	59				C_rel_^a^	0.040	0.098	0.206	0.389	0.677
Typical Molecules ^c^	59	yes	part	no	C_rel_^a^	0.022	0.051	0.106	0.196	0.340
Dirac-Fock Atoms	61	part	no	no	C_rel_^b^	0.024	0.059	0.126	0.243	0.434
Dirac-Fock Atoms	54–58	part	no	no		0.06	0.13	0.25	0.45	0.75

^a^ C_rel_ = (2.198 × 10^−7^) I_nr_^2.178^. ^b^ (6.55 × 10^−7^) I_nr_^2.0569^. ^c^ B_2_H_6_, CH_4_, NH_3_, H_2_O, HF. It can be seen that the relativistic corrections reported by Maruani et al. [61] are slightly larger than C_rel_ of our earlier empirical fit [59] and approximately half of those reported by Bellafont at al [54,55,56,57,58]. It would be ideal if one can apply a relativistic procedure capable of giving accurate results for two-electron ions to the typical molecules B_2_H_6_, CH_4_, NH_3_, H_2_O, and HF. Until then, we shall remain consistent and continue to use our C_rel_.

**Table 4:** Carbon core-electron binding energies (in eV).

Case	Obs	ΔPW86PW91	ΔTPSS
**C**H_2_=C(CH_3_)_2_	289.83	289.88	290.66
**C_α_** in pyrrole	289.96	289.92	289.76
**C**H_2_=CHCH_3_	290.25	290.24	290.61
**C_m_** in C_6_H_5_F	290.54	290.49	290.57
**C_p_** in C_6_H_5_F	290.54	290.69	290.38
CH_2_=**C**(CH_3_)_2_	290.65	290.76	290.52
CH_2_=C(**C**H_3_)_2_	290.69	290.78	290.66
CH_2_=**C**HCH_3_	290.73	290.79	290.82
**C_β_** in pyrrole	290.77	290.75	290.80
CH_2_=CH**C**H_3_	290.81	290.95	290.17
**C_o_** in C_6_H_5_F	290.87	290.63	290.52
**C**H_4_	290.91	290.95	290.86
**C**_2_ in *p*-C_6_H_4_F_2_	290.99	290.92	290.89
**C**H_3_COOH	291.55	291.51	291.83
**C**H_3_OCH_3_	292.34	292.21	292.17
**C**H_3_OH	292.42	292.54	292.45
CH_3_**C**N	292.45	292.75	292.70
**C_1_** in C_6_H_5_F	292.70	292.75	292.55
**C**_1_ in *p*-C_6_H_4_F_2_	292.95	292.86	292.74
**C**H_3_CN	292.98	292.84	292.62
H**C**N	293.40	293.56	293.46
H_2_**C**O	294.47	294.53	294.49
CH_3_**C**OOH	295.38	295.06	294.99
**C**O	296.21	296.26	296.32
**C**H_2_F_2_	296.40	296.08	296.20
**C**O_2_	297.69	297.28	297.30
**C**F_4_	301.90	301.14	301.34
**C**_p_ in aniline	289.85	289.85	290.02
**C**_o_ in aniline	289.95	289.97	289.89
**C**_m_ in aniline	290.25	290.17	290.03
**C**_1_ in aniline	291.29	291.37	291.17
**C**_p_ in toluene	290.1	290.24	290.02
**C**_o_ in toluene	290.2	290.19	289.89
**C**_m_ in toluene	290.4	290.31	290.03
**C**_1_ in toluene	290.9	290.49	290.17
**C**_p_ in phenol	290.2	290.24	289.94
**C**_o_ in phenol	290.2	290.47	290.21
**C**_m_ in phenol	290.6	290.53	290.25
**C**_1_ in phenol	292.0	292.12	291.84
Average deviation	(0)	−0.03	−0.11
Average absolute deviation	(0)	0.14	0.23
AD for **Δ**PW86PW91 + 0.08		+0.05	
AAD for **Δ**PW86PW91 + 0.08		0.16	

**Table 5:** Core-electron binding energies (in eV) of N, O, and F. AAD (n) means average absolute deviation for n cases.

Case	Obs	ΔPW86PW91	ΔTPSS [56]	ΔB3LYP [56]	QsGW [62]
N in pyridine	404.88	404.76	404.43		
NH_3_	405.56	405.77	405.52	405.32	407.84
N in pyrrole	406.15	406.37	405.96		
HCONH_2_	406.38	406.55			
HCN	406.78	406.94	406.67	406.66	408.78
NNO	408.71	408.59			
N_2_	409.98	410.02			
NNO	412.59	412.47			
AAD(4) for N1s	(0)	0.18	0.20		
AAD(8) for N1s	(0)	0.14			
HCONH_2_	537.77	537.78			
CH_3_OCH_3_	538.74	538.71	538.04		
CH_3_OH	539.48	539.15	538.59		
H_2_CO	539.48	539.48	539.03		
H_2_O	539.90	540.01	539.45	539.39	542.13
CO_2_	541.28	541.36	540.96		
CO	542.55	542.72	542.21	542.10	
AAD(6) for O1s	(0)	0.12	0.52		
AAD(7) for O1s	(0)	0.10			
HF	694.23	694.28	693.53		
CH_2_F_2_	693.65	693.71	692.89		
CF_4_	695.56	695.38	694.58		
F_2_	696.69	696.52	695.89		
AAD(4) for F1s	(0)	0.04	0.81		

**Table 6:** Ultraviolet excitation spectrum of indole vapor: energies in eV (*f*-values in parentheses, unless otherwise specified).

State	Experiment								Theory				
	1963 ^a^	1970 ^b^	1977 ^c^	1995 ^d^	1996 ^e^	2007 ^f^	2011 ^g^	2015 ^h^	CASPT2	CASPT2	CCR(3)	RASPT2	DFT ^k^
									1996 ^e^	2000 ^i^	2011 ^g^	2017 ^j^	2020 ^k^
2 ^1^A’	4.37	4.32		4.35 m	4.37 (0.045)	4.37	4.37	4.32	4.43 (0.050)	4.43 (0.050)	4.76 (0.038)	4.31 (0.38)	4.45 (0.0543)
3 ^1^A’		4.77		4.67 s	4.77 (0.123)	4.63	4.79		4.73 (0.081)	4.73 (0.081)	5.12 (0.1018)	4.64 (0.23)	4.64 (0.0187)
1 ^1^A”		4.86				4.784.87	4.90		4.85 (0.001)		5.02 (0.0022)	5.84 (0.14)	5.34 (0.0015)
4 ^1^A’					5.27				5.21 (0.004)	5.84 (0.458)		5.96 (0.11)	5.62 (0.1761)
2 ^1^A”								5.71	5.33 (0.003)			6.01 (0.36)	5.75 (0.0011)
3 ^1^A”									5.36 (0.001)			6.17 (0.54)	5.76 (0.0010)
5 ^1^A’					5.55	5.90	6.02		5.65 (0.002)	6.16 (0.003)		6.42 (0.10)	5.87 (0.3682)
4 ^1^A”									5.37 (0.002)			6.55 (0.11)	6.08 (0.0018)
6 ^1^A’			6.04 ^h^		6.02 (~0.6)				5.84 (0.458)	6.44 (0.257)		7.40 (0.88)	6.08 (0.0571)
5 ^1^A”									5.81 (0.001)			7.39 (0.17)	6.17 (0.0019)
7 ^1^A’			6.34 ^h^		6.35				5.94 (0.012)	6.71 (0.138)			6.38 (0.2593)

^a^ Hollas [17]. ^b^ Strickland et al. [18]. ^c^ Lami [19,20]. ^d^ Ilich, in Ar matrix [21]. ^e^ Serrano-Andres and Roos [22]. ^f^ Borisevich and Raichenok [23]. ^g^ Livingston et al. [24]. ^h^ Kumar et al. [25]. ^i^ Borin and Serrano-Andres [26,27]. ^j^ Giussani et al. [28]: transition dipoles in parentheses. ^k^ This work: TDDFT using V_xc_ = SAOP/aug-et-pVQZ.

**Table 7:** UV absorption spectrum of 7-azaindole vapor: energies in eV (f-values in parentheses).

State	Experiment					Theory				
						INDO/SI	CASPT2	CASPT2	TDDFT	This Work ^i^
	1984 ^a^	1984 ^b^	1989 ^c^	1995 ^d^	2018 ^e^	1995 ^d^	2000 ^f^	2001 ^g^	2016 ^h^	2020
2 ^1^A’	4.29	4.15	4.29	4.28	4.29	4.28 (0.17)	4.22 (0.043)	4.22 (0.043)	4.56	4.20 (0.0456)
1 ^1^A”							5.27 (0.008)	5.27 (0.008)	4.84	4.60 (0.0018)
3 ^1^A’		4.49		4.49		4.55 (0.09)	4.49 (0.072)	4.49 (0.072)		4.62 (0.0571)
2 ^1^A”										5.64 (0.0002)
4 ^1^A’				5.76			5.77 (0.065)	5.77 (0.065)		5.69 (0.3733)
3 ^1^A”										5.82 (0.0034)
5 ^1^A’				5.99			5.93 (0.148)	5.93 (0.148)		6.02 (0.0518)
4 ^1^A”										6.10 (0.0000)
5 ^1^A”										6.25 (0.0029)
6 ^1^A’							6.26 (0.377)	6.26 (0.377)		6.36 (0.0718)
6 ^1^A”										6.47 (0.0018)
7 ^1^A’							6.46 (0.079)	6.46 (0.079)		6.51 (0.0763)
7 ^1^A”										6.52 (0.0085)
8 ^1^A”										6.52 (0.0008)
8 ^1^A’							6.71 (0.265)	6.71 (0.265)		6.64 (0.2339)

^a^ Fuke et al. [29]. ^b^ Bulska et al., in ethanol [30]. ^c^ Hassan and Hollas [31]. ^d^ Ilich, in Ar matrix [21]. ^e^ Sukhodola [32]. ^f^ Serrano-Andres and Borin [27]. ^g^ Serrano-Andres et al. [33]. ^h^ Ten et al. [34]. ^i^ This work: TDDFT using V_xc_ = SAOP/aug-et-pVQZ.

**Table 8:** UV excitation spectrum of n-azaindole vapor, *n* = 4, 5, and 6: energies in eV (*f*-values in parentheses).

State	*n* = 4	*n* = 5	*n* = 6
2 ^1^A’	4.23 (0.0383)	4.46 (0.0479)	4.40 (0.0521)
3 ^1^A’	4.62 (0.0646)	4.85 (0.0079)	4.82 (0.0211)
4 ^1^A’	5.66 (0.2827)	5.67 (0.1402)	5.80 (0.1531)
5 ^1^A’	5.93 (0.0382)	5.86 (0.1474)	5.86 (0.2207)
6 ^1^A’	5.98 (0.1296)	5.94 (0.1817)	5.97 (0.0103)
7 ^1^A’	6.43 (0.1104)	6.44 (0.2553)	6.21 (0.1286)
8 ^1^A’	6.47 (0.0378	6.49 (0.0751)	6.47 (0.3539)
1 ^1^A”	4.26 (0.0016)	4.33 (0.0004)	4.33 (0.0012)
2 ^1^A”	5.27 (0.0005)	5.18 (0.0025)	5.29 (0.0006)
3 ^1^A”	5.62 (0.0017)	5.49 (0.0017)	5.62 (0.00010
4 ^1^A”	5.93 (0.0008)	6.03 (0.0001)	6.06 (0.0013)
5 ^1^A”	6.12 (0.0020)	6.16 (0.0007)	6.09 (0.0015)
6 ^1^A”	6.16 (0.0012)	6.32 (0.0077)	6.31 (0.0001)
7 ^1^A”	6.43 (0.0001	6.35 (0.0018)	6.41 (0.0034)

**Table 9:** Vertical ionization energies (in eV) of indole vapor.

MO	2020				2020			1976	2014
	This Work				Plekan et al. ^a^			Gusten ^b^	Chrostowska ^c^
	HAM/3 ^d^	mKT ^e^	DFT ^f^	Ave.	Obs	P3+	Ave.	Obs	Obs
5π	8.29 (0.0517)	9,23	7.78		7.90	7.91		7.91	7.9
4π	8.85 (0.0467)	9.65	8.24		8.32	8.25		8.37	8.5
3π	9.99 (0.0418)	10.96	9.81		9.82	9.88		9.78	9.9
26σ	11.93 (0.0394)	12.06	11.35		10.97	11.68		11.03	11.05
2π	11.12 (0.0450)	12.30	11.38		11.55	11.29		11.52	11.45
25σ	12.40 (0.0485)	12.58	11.97		12.20	12.27		12.26	12.25
24σ	13.26 (0.0572)	13.52	12.93		13.02	13.29		12.72	13.0
23σ	13.55 (0.0402)	14.00	13.51		(13.7)	13.88		13.16	
1π	13.24 (0.0535)	14.49	13.75		13.80	13.66		13.77	
22σ	13.97 (0.0463)	14.46	14.02		(14.1)	14.37		(14.04)	
21σ	14.44 (0.0617)	14.68	14.08		14.25	14.52		14.25	
20σ	15.06 (0.0710)	15.54	15.12		15.30	15.58		15.05	
19σ	15.55 (0.1286)	15.86	15.45		15.80	15.88		15.33	
18σ	16.61 (0.1039)	17.31	17.04		17.00	17.48		17.03	
17σ	17.50 (0.2076)	18.38	18.26		(18.2)				
16σ	18.48 (0.2547)	18.73	18.68		18.52				
15σ	18.74 (0.1813)	19.45	19.42		19.25				
14σ	21.84 (0.4390)	21.83	22.06						
13σ	22.51 (0.4648)	22.29	22.58						
12σ	23.95 (0.5046)	23.50	23.91						
11σ	25.96 (0.5796)	24.94	25.48						
10σ	30.18 (0.6925)	28.62	29.43						
C8		289.72	289.72	289.85	289.89	289.49	289.61		
C7		289.82	289.77			289.54			
C3		289.67	289.78			289.55			
C9		289.83	289.79			289.57			
C6		289.97	290.00			289.76			
C4		289.92	290.02			289.77			
C5		290.76	(290.76) ^g^	290.78	290.86	290.61	290.64		
C2		290.78	290.79			290.66			
N1			406.00		405.82	405.45			

^a^ Plekan et al. [35]: P3+ for outer-valence electrons and ΔB3LYP for core electrons. ^b^ Gusten et al. [74]. ^c^ Chrostowska et al. [75]. ^d^ Relative intensity for XPS in parentheses, based on the Gelius model. ^e^ mKT + shift of 16.67 eV for core electrons. ^f^ ΔPBE0(SAOP)/et-pVQZ//B3LYP/6-31G(d) for valence electrons; ΔPW86xPW91c + C_rel_ for core electrons. ^g^ Shifted mKT.

**Table 10:** Predicted vertical ionization energies (in eV) of azaindoles ^a^.

MO	4-Azaindole	5-Azaindole	6-Azaindole	7-Azaindole
5π	8.29 (0.0552)	8.16 (0.0499)	8.25 (0.0572)	8.22 (0.0530)
4π	8.61 (0.0566)	9.09 (0.0667)	8.81 (0.0492)	8.67 (0.0552)
3π	10.44 (0.0429)	10.19 (0.0408)	10.33 (0.0511)	10.58 (0.0511)
2π	12.18 (0.0655)	12.34 (0.0636)	12.30 (0.0631)	12.04 (0.0601)
1π	14.15 (0.0528)	14.18 (0.0530)	14.20 (0.0533)	14.27 (0.0549)
26σ	9.18 (0.2224)	9.16 (0.2354)	9.22 (0.2353)	9.57 (0.2135)
25σ	12.44 (0.0493)	11.92 (0.0446)	12.04 (0.0546)	12.52 (0.0443)
24σ	13.06 (0.0508)	13.24 (0.0554)	12.79 (0.0453)	~12.9 ^b^ (0.089)
23σ	13.41 (0.0802)	13.98(0.0693)	14.08 (0.0546)	13.22 (0.0751)
22σ	14.03 (0.0637)	14.29 (0.0501)	14.13 (0.0444)	14.06 (0.0575)
21σ	14.73 (0.0502)	~14.7 ^b^ (0.0504)	14.73 (0.0593)	14.73 (0.0542)
20σ	15.63 (0.0683)	15.21 (0.0840)	15.39 (0.0813)	15.41 (0.0640)
19σ	15.96 (0.1277)	16.03 (0.1192)	15.90 (0.1275)	15.90 (0.1173)
18σ	17.31 (0.1197)	17.57 (0.1003)	17.60 (0.1058)	17.41 (0.1203)
17σ	18.66 (0.2008)	18.82 (0.212)	18.79 (0.2240)	18.82 (0.2020)
16σ	19.22 (0.2565)	19.21 (0.2483)	19.18 (0.2619)	19.18 (0.2450)
15σ	20.11 (0.2364)	20.17 (0.2310)	20.11 (0.2213)	20.08 (0.2544)
14σ	22.60 (0.4581)	22.56 (0.4403)	~22.8 ^b^ (0.4536)	22.65 (0.4506)
13σ	23.77 (0.4929)	~23.4 ^b^ (0.5260)	23.30 (0.4915)	23.37 (0.4881)
12σ	24.57 (0.5415)	24.04 (0.5327)	24.69 (0.5583)	24.85 (0.5656)
11σ	28.12 (0.6945)	28.20 (0.7018)	28.19 (0.6963)	27.98 (0.6628)
10σ	29.75 (0.7095)	~29.9 ^b^ (0.6993)	29.86 (0.7016)	29.84 (0.7255)
	C3 (289.77) ^c^	C3 290.18	C4 290.28	C5 290.05
	C6 290.07	C9 290.34	C3 290.34	C3 290.06
	C7 290.57	C7 (290.37) ^c^	C9 290.70	C9 290.34
	C5 290.61	C6 290.59	C5 290.71	C4 290.35
	C9 290.80	C4 290.71	C7 291.07	C6 290.67
	C8 (291.01) ^c^	C2 291.17	C2 291.34	C2 291.05
	C2 291.05	C6 (291.37) ^c^	C8 (291.23) ^c^	C8 (291.54) ^c^
	N4 404.06	N5 404.01	N6 404.21	N7 404.36
	N1 406.30	N1 406.39	N1 406.57	N1 406.13

^a^ ΔPBE0(SAOP)/et-pVQZ//B3LYP/6-31G(d) for valence electrons; ΔPW86xPW91c + C_rel_ for core electrons; approximate relative intensities for XPS (based on the Gelius model) in parentheses. ^b^ Estimated, for nonconvergent cation. ^c^ mKT + shift.

**Table 11:** Predicted X-ray emission spectrum of indole and 7-azaindole: ΔE in eV, *f*-value in parentheses.

	Indole	7-Azaindole	
	Core Hole @ N1	N1	N7
5π	398.22 (0.0061)	397.91 (0.0040)	396.14 (0.0019)
4π	397.76 (0.0040)	397.46 (0.0082)	395.69 (0.0028)
3π	396.19 (0.0037)	395.55 (0.0027)	393.78 (0.0076)
2π	394.62 (0.0064)	394.09 (0.0082)	392.32 (0.0084)
1π	392.25 (0.0019)	**391.86 (0.0111)**	390.09 (0.0042)
26σ	394.65 (0.0005)	396.56 (0.0007)	**394.79 (0.0252)**
25σ	394.03 (0.0009)	393.61 (0.0004)	391.84 (0.0033)
24σ	393.07 (0.0033)	393.23 (0.0023)	391.46 (0.0038)
23σ	302.49 (0.0004)	392.91 (0.0027)	391.14 (0.0026)
22σ	**391.98 (0.0143)**	392.07 (0.0024)	390.30 (0.0020)
21σ	391.92 (0.0079)	391.40 (0.0071)	389.63 (0.0033)
20σ	390.88 (0.0006)	390.72 (0.0036)	388.95 (0.0043)
19σ	390.55 (0.0084)	390.23 (0.0058)	388.46 (0.0010)
18σ	388.96 (0.0068)	388.72 (0.0076)	386.95 (0.0030)
17σ	387.74 (0.0051)	387.31 (0.0095)	385.54 (0.0010)
16σ	387.32 (0.0072)	386.95 (0.0027)	385.18 (0.0066)
15σ	386.58 (0.0065)	386.05 (0.0057)	384.28 (0.0002)
14σ	383.94 (0.0022)	383.48 (0.0038)	381.71 (0.0029)
13σ	383.42 (0.0024)	382.78 (0.0011)	380.99 (0.0002)
12σ	382.09 (0.0016)	381.28 (0.0006)	379.51 (0.0001)
11σ	380.52 (0.0003)	378.15 (0.0003)	376.38 (0.0017)
10σ	376.57 (0.0005)	376.29 (0.0005)	374.52 (0.0004)

**Table 12:** Predicted X-ray emission spectrum of azaindoles: ΔE in eV, *f*-value in parentheses.

	4-Azaindole		5-Azaindole		6-Azaindole	
	N1	N4	N1	N5	N1	N6
5π	398.01 (0.0039)	395.77 (0.0038)	398.23 (0.0047)	395.85 (0.0002)	398.32 (0.0088)	395.96 (0.0018)
4π	397.69 (0.0092)	395.45 (0.0018)	397.30 (0.0076)	394.92 (0.0099)	397.76 (0.0008)	395.40 (0.0052)
3π	395.86 (0.0004)	**393.62 (0.0261)**	396.20 (0.0027)	393.82 (0.0002)	396.24 (0.0056)	393.88 (0.0032)
2π	394.12 (0.0002)	391.88 (0.0053)	394.05 (0.0063)	**391.67 (0.0130)**	394.27 (0.0061)	**391.91 (0.0128)**
1π	**392.15 (0.0120)**	389.91 (0.0022)	**392.21 (0.0132)**	389.83 (0.0010)	**392.37 (0.0131)**	390.01 (0.0013)
26σ	397.12 (0.0005)	394.88 (0.0055)	397.23 (0.0001)	**394.85 (0.0276)**	397.35 (0.0003)	**394.99 (0.0273)**
25σ	393.86 (0.0089)	**391.62 (0.0110)**	394.47 (0.0003)	392.09 (0.0046)	394.53 (0.0014)	392.17 (0.0044)
24σ	393,24 (0.0025)	391.00 (0.0038)	393.15 (0.0035)	390.77 (0.0010)	393.76 (0.0005)	391.42 (0.0025)
23σ	392.89 (0.0021)	390.65 (0.0011)	392.41 (0.0013)	390.03 (0.0030)	392.49(0.0015)	390.13 (0.0032)
22σ	392.27 (0.0020)	390.03 (0.0020)	392.10 (0.0035)	389.72 (0.0024)	392.44(0.0015)	390.08 (0.0005)
21σ	391.57 (0.0070)	389.33 (0.0035)	391.69 (0.0073)	389.31 (0.0027)	391.84 (0.0091)	389.48 (0.0032)
20σ	390.67 (0.0002)	388.43 (0.0037)	391.18 (0.0071)	388.80 (0.0044)	391.18 (0.0018)	388.82 (0.0058)
19σ	**390.34 (0.0104)**	388.10 (0.0029)	390.36 (0.0064)	387.98 (0.0028)	390.67 (0.0085)	388.31 (0.0007)
18σ	388.99 (0.0073)	386.75 (0.0004)	388.82 (0.0067)	386.44 (0.0022)	388.97 (0.0071)	386.61 (0.0043)
17σ	387.64 (0.0085)	385.40 (0.0017)	387.57 (0.0084)	385.19 (0.0006)	387.78 (0.0074)	385.42 (0.0006)
16σ	387.08 (0.0034)	384.84 (0.0053)	387.18 (0.0044)	384.80 (0.0036)	387.39 (0.0054)	385.03 (0.0004)
15σ	386.19 (0.0058)	383.95 (0.0019)	386.22 (0.0072)	383.84 (0.0022)	386.46 (0.0057)	384.10 (0.0044)
14σ	383.70 (0.0034)	381.46 (0.0011)	383.83 (0.0020)	381.45 (0.0025)	383.77 (0.0009)	381.41 (0.0021)
13σ	382.53 (0.0018)	380.29 (0.0010)	382.99 (0.0019)	380.61 (0.0004)	383.27 (0.0039)	380.91 (0.0006)
12σ	381.73 (0.0009)	379.49 (0.0002)	382.35 (0.0013)	379.97 (0.0002)	381.88 (0.0009)	379.52 (0.0001)
11σ	378.18 (0.0000)	375.94 (0.0019)	378.19 (0.0000)	375.81 (0.0019)	378.38 (0.0000)	376.02 (0.0019)
10σ	376.55 (0.0005)	374.31 (0.0001)	376.49 (0.0005)	374.11 (0.0000)	376.71 (0.0005)	374.35 (0.0000)

## Data Availability

Data availability on request.

## Supplementary Materials

The following are available online: the optimized Cartesian coordinates of the five molecules.

## Appendix A

The geometry of both methanal and hydrogen peroxide were optimized by popular methods using various basis sets. The results of such test calculations are presented in Table A1 and Table A2. It can be seen that the dependence of the calculated CEBEs is quite small in both cases.

**Table A1:** Summary of DF T calculation on H_2_CO: bond lengths in angstrom, angles in degree, rotational constants and their average absolute deviation from experiment in MHz, and core-electron binding energies in eV. Bold-face type indicates best agreement with the experiment.

Method	R(CO)	R(CH)	<OCH	A	B	C	AAD	OK	Dev	CK	Dev
B3LYP/6-31G(d)	1.2066	1.1105	122.38	285,068	38,634	34,023	1108	539.56	0.08	294.57	0.10
B3LYP/6-311 + G(d,p)	1.2019	1.1080	122.04	284,214	38,999	34,293	903	539.56	0.08	294.55	0.08
B3LYP/6-311 + G(2d,p)	1.2003	1.1075	121.89	283,545	39,135	34,389	**757**	539.56	0.08	294.55	0.08
MP2/cc-pVTZ	1.2102	1.1004	121.95	287,589	38,603	34,033	1962	539.54	0.06	294.54	0.07
CCSD/cc-pVTZ	1.2028	1.1012	121.94	287,122	39,022	34,353	1900	539.55	0.07	294.53	0.06
CCSD(T)/cc-pVTZ	1.2096	1.1030	121.88	285,816	38,631	34,031	1361	539.54	0.06	294.55	0.08
Expt	1.2078	1.1161	116.52	281,964	38,832	34,002	(0)	539.48	(0)	294.47	(0)

**Table A2:** Summary of DFT calculation on HOOH: bond lengths in angstrom, angles in degree, rotational constants and their average absolute deviation from experiment in MHz, and core-electron binding energies in eV. Bold-face type indicates best agreement with the experiment.

Method	R(OO)	R(OH)	<HOO	<HOOH	A	B	C	AAD	CEBE	Dev
B3LYP/6-31G(d)	1.4557	0.9737	99.68	118.60	297,104	26,414	25,366	4799	540.89	0.09
B3LYP/6-311+G(d,p)	1.4538	0.9672	100.50	120.84	303,402	26,466	25,364	2717	540.89	0.09
B3LYP/6-311+G(2d,p)	1.4516	0.9682	100.55	115.67	302,390	26,449	25,518	3100	540.90	0.10
MP2/cc-pVTZ	1.4498	0.9643	99.36	114.13	301,579	26,598	25,704	3482	540.92	0.12
CCSD/cc-pVTZ	1.4406	0.9610	100.27	113.09	305,937	26,836	25,979	**2200**	540.93	0.13
CCSD(T)/cc-pVTZ	1.4578	0.9640	99.55	113.93	302,158	26,301	25,435	3100	540.91	0.11
Expt	1.475	0.950	94.8	119.8	310,465	25,950	24,793	(0)	540.8	(0)
