# Supplementary material for: Computational Study of the Electron Spectra of Vapor-Phase Indole and Four Azaindoles
Source: Molecules. 2021 Mar 30;26(7):1947. doi: 10.3390/molecules26071947 (PMC8037839; doi:10.3390/molecules26071947)
Supplement: Supplementary file 1 [file molecules-26-01947-s001.pdf]

indole //B3LYP/6-31G*
N -1.095851 1.556298 0.000000
C -2.253298 0.798644 0.000000
C -1.929484 -0.532149 0.000000
C -0.494651 -0.619015 0.000000
C 0.000000 0.716386 0.000000
C 1.368353 1.007590 0.000000
C 2.252682 -0.064885 0.000000
C 1.786696 -1.396104 0.000000
C 0.427268 -1.680706 0.000000
H 0.078287 -2.710327 0.000000
H 2.506489 -2.210168 0.000000
H 3.322300 0.126721 0.000000
H 1.728924 2.033250 0.000000
H -2.629678 -1.356285 0.000000
H -3.221253 1.280676 0.000000
H -1.059502 2.563488 0.000000

4-azaindole //B3LYP/6-31G(d)
N 1.129851 1.519810 0.000000
C 2.249604 0.704234 0.000000
C 1.869470 -0.611720 0.000000
N -0.415397 -1.671183 0.000000
C -1.711772 -1.364048 0.000000
C -2.227153 -0.051620 0.000000
C -1.360184 1.036328 0.000000
C 0.000000 0.732370 0.000000
C 0.434090 -0.627380 0.000000
H 1.145398 2.527810 0.000000
H 3.238353 1.141831 0.000000
H 2.521634 -1.473082 0.000000
H -2.401669 -2.206751 0.000000
H -3.302183 0.101003 0.000000
H -1.727049 2.059820 0.000000

5-azaindole //B3LYP/6-31G(d)
N 1.105134 1.538650 0.000000
C 2.251726 0.756688 0.000000
C 1.905240 -0.566643 0.000000
C -0.497569 -1.639654 0.000000
N -1.805825 -1.394231 0.000000
C -2.214941 -0.105609 0.000000
C -1.370170 0.997853 0.000000
C 0.000000 0.721191 0.000000
C 0.468574 -0.620717 0.000000
H 1.088473 2.546788 0.000000
H 3.227462 1.222377 0.000000
H 2.589290 -1.403806 0.000000
H -0.199756 -2.688078 0.000000
H -3.293090 0.043125 0.000000

H -1.764700 2.010005 0.000000

6-azaindole //B3LYP/6-31G(d)
N 1.100237 1.536109 0.000000
C 2.245809 0.766846 0.000000
C 1.913483 -0.564578 0.000000
C -0.480592 -1.660358 0.000000
C -1.818764 -1.290384 0.000000
N -2.267394 -0.013703 0.000000
C -1.371899 0.969893 0.000000
C 0.000000 0.701839 0.000000
C 0.480750 -0.635577 0.000000
H 1.074237 2.543935 0.000000
H 3.219034 1.238542 0.000000
H 2.608364 -1.392745 0.000000
H -0.195825 -2.708783 0.000000
H -2.593397 -2.054634 0.000000
H -1.755040 1.990758 0.000000

7-azaindole //B3LYP/6-31G(d)
N 1.109033 1.496005 0.000000
C 2.246502 0.710363 0.000000
C 1.896633 -0.615874 0.000000
C -0.524642 -1.662449 0.000000
C -1.854592 -1.252993 0.000000
C -2.170516 0.118944 0.000000
N -1.263618 1.101378 0.000000
C 0.000000 0.680814 0.000000
C 0.462626 -0.667935 0.000000
H 1.071610 2.503932 0.000000
H 3.226031 1.167822 0.000000
H 2.582303 -1.452022 0.000000
H -0.263395 -2.717744 0.000000
H -2.658273 -1.983159 0.000000
H -3.212255 0.434267 0.000000
